# Tree diversity and carbon stocks in agroforestry systems in northern Ethiopia

**DOI:** 10.1186/s13021-021-00174-7

**Published:** 2021-05-04

**Authors:** Ashenafi Manaye, Berihu Tesfamariam, Musse Tesfaye, Adefires Worku, Yirga Gufi

**Affiliations:** 1 Tigray Institute of Policy Studies, Mekelle, Ethiopia; 2 Mekele Environment and Forest Research Center, Mekelle, Ethiopia; 3 Central Ethiopia Environment and Forest Research Center, Addis Ababa, Ethiopia; 4 Ethiopian Environment and Forest Research Institute, P.O.Box 24536, code 1000 Addis Ababa, Ethiopia

**Keywords:** Boundary planting, Home garden, Parkland, Resilience, Woodlot

## Abstract

**Background:**

Agroforestry (AF) is an ancient tradition in Ethiopian dryland farming system. Several studies have examined system design, soil fertility management and system interactions, but the biodiversity and mitigation aspects of climate change have received less focus. We assessed the diversity of woody species, biomass carbon (C), and soil organic carbon (SOC) stock associated with indigenous dryland AF practices. A total of 197 smallholder farmers representing four AF practices (home garden, parkland, boundary plantation, and woodlot) from lowland, midland, and highland areas were systematically selected. The inventory of woody species was carried out on each farm's randomly formed plot.

**Results:**

We identified a total of 59 woody species representing 48 genera and 32 families. Shannon diversity index (H') was highest in home garden and parkland AF, while woodlots had the highest mean total stock of biomass C (31 Mg C ha^−1^). C stocks for smallholding systems (total biomass C and SOC 0–60 cm) ranged from 77–135 Mg ha^−1^. Total biomass C stocks were significantly correlated with abundance (Spearman r = 0.333; p = 0.002) but biomass components were not significantly correlated with H'. SOC soil depth stock (0–60 cm) was positively and significantly associated with H' (Spearman r = 0.291 & 0.351; p < 0.01).

**Conclusions:**

We report greater species richness in home garden and parkland AF systems than in woodlots. While parkland AF produce lower biomass and SOC stock relative to other AF systems. The strategic use of home gardens and boundary planting can improve tree diversity and carbon storage in Ethiopian dryland ecosystems.

## Background

In addition to its global implications, climate change poses a particularly serious threat to developing countries [1–3]. Climate trends directly and indirectly affects both food production and the capacity of natural ecosystems to provide products and services. Sub-Saharan Africa (SSA) has pronounced vulnerabilities linked to endemic poverty and large rural populations whose livelihoods depend on agriculture and ecosystem services that are highly sensitive to rainfall variability [2, 4, 5].

This is a major concern in Ethiopia, where much of the population is chronically food insecure [4, 6, 7]. The northern part of the country (study region) has been identified as particularly climate change vulnerable [8]. Rural populations rely on natural forests for food and income, leading to degradation, and have migrated to marginal and vulnerable areas prone to land loss, drought, pest, and disease outbreaks.

Like several other SSA countries, Ethiopia has a high potential for climate change adaptation and mitigation via agriculture, forestry, and other land use (AFOLU)-based pathways, but this requires balancing conflicting social and ecological demands [2].

Despite a thousand-year history of incorporating trees and shrubs into agricultural land management techniques in Ethiopia [9], however, indigenous, and traditional AF knowledge has been inconsistently formalized and is often excluded from national-scale policy processes. This knowledge is a potentially significant untapped resource: by enhancing carbon stocks and potentially improving agricultural productivity, AF offers a potential solution that could help Ethiopia meet its reforestation and climate-smart agriculture commitments while attending to the needs of vulnerable groups [9, 10]**.**

Despite the important contribution of AF systems to climate change adaptation and mitigation [1, 4, 11–13], not all AF systems are designed to be efficient and widely implemented. Key knowledge gaps include which system works better where, for whom, and under which conditions with climate resilience presenting additional complications [2, 14]**.** In order to provide an empirical foundation to facilitate the integration of AF into current policy and practices, this study examines how indigenous dryland AF practices in the Tigray region impact on woody plant diversity and carbon storage.

## Methods

### Study site

**Fig. 1:**
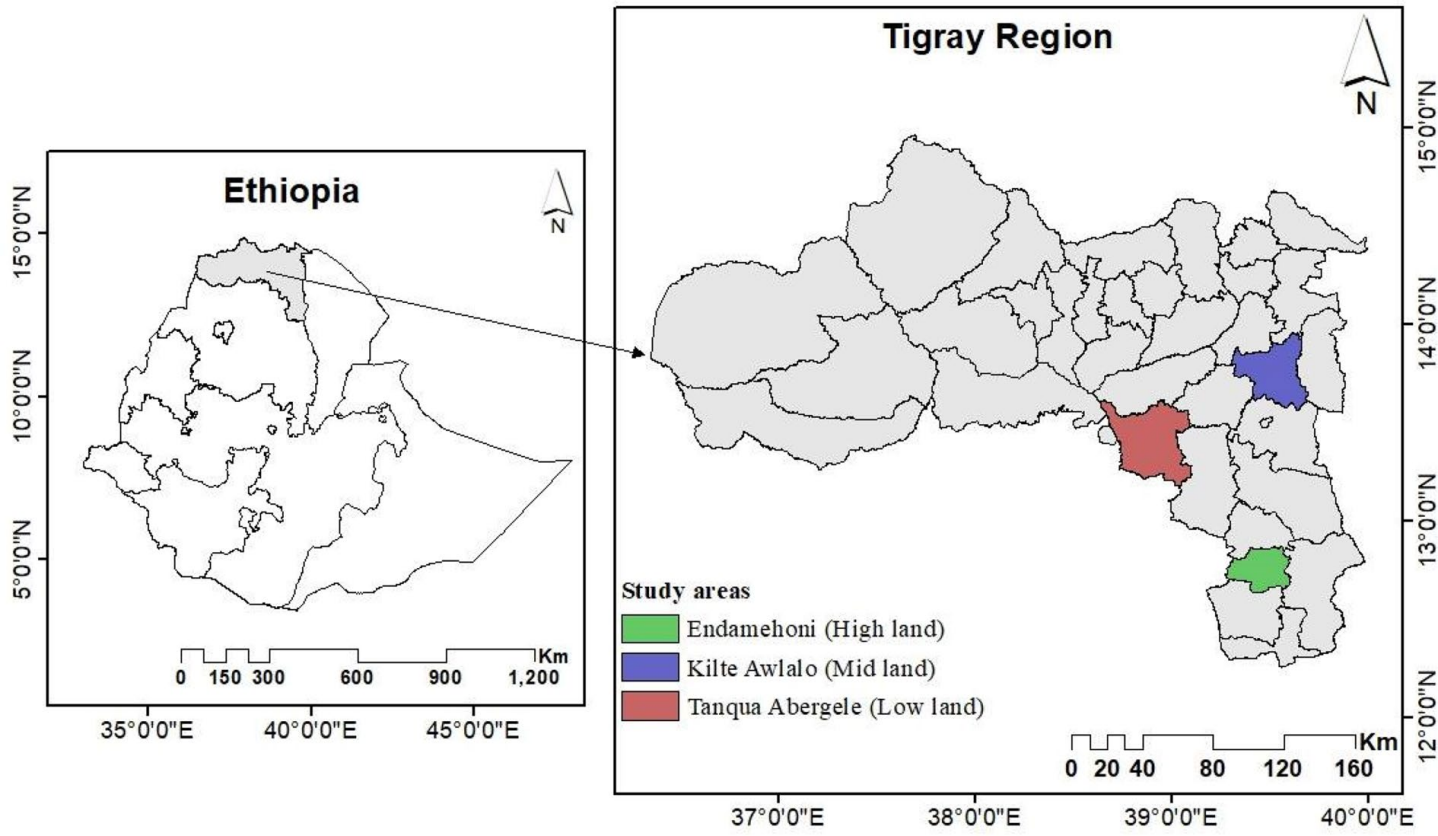
Location of the study area

The study was conducted in three agroecologies (lowland, midland and highland) of the regional state of Tigray, which are geographically located between 12° and 15° N latitude and 36° 30′–40° 30′ E longitude (Fig. 1).

Table 1 shows the mean annual rainfall, and monthly minimum and maximum temperature of the study area (based on the climate data from years 2000–2014). Except for Tanqu Abergele (Abyi Adi station), both Endamehoni (Maychew station) and Klilte Awlalo (Wukiro station) report a bimodal rain fall pattern. A description of the three agroecologies in the study area is given in Table 1.

**Table 1:** Characteristics of the three Agroecology indigenous agroforestry systems in the Tigray Region, Northern Ethiopia

Characteristics	Lowland	Midland	Highland
Altitude (m a.s.l)	1300–1500	1930 to 2500	1,800 to 3,250
Mean annual rain falls (mm)	400 to 600	397 to 903	478 to 956
Average minimum and maximum T^o^ (^o^C)	14.3 to 29.9	11.1 to 28.0	10.2 to 22.5
Dominant soil type	Cambisols	Leptosols	Leptosols
Textural Class	Sandy loam	Sandy loam	Sandy loam
Average pH ± sd	7.9 ± 0.4	7.3 ± 0.5	7.3 ± 0.4
Mean size of farms studied (ha) ± sd	1.62 ± 0.76	0.89 ± 0.78	0.76 ± 0.49
Major trees	*Ziziphus spina-christi, Acacia etbaica; Acacia seyal*	*Faidherbia albida, Eucalyptus camaldulensis, Faidherbia albida, Acacia saligna*	*Eucalyptus globulus, Acacia abyssinica, Acacia saligna, Olea a fricana, Psidium guajava,*
Major food and cash crops	*Zea mays, Sorghum bicolour, Eragrostis teff, Linum usitatissimum and Eleusine coacan*	*Triticum aestivum, Eragrostis teff, Zea mays, Eleusine coacana*	*Hordeum vulgare, Triticum aestivum and Zea mays*

### Sampling design and methods of data collection

First, preliminary survey was performed to identify the characteristics of each AF (spatial distribution, their function and structure) and specific sites featuring an AF practices across different agroecologies. A multistage sampling technique was employed to collect the data. The agroecologies were purposively selected to capture both the range of AF practices and the agroecological variation across the region, which encompasses highland, lowland, and midland areas.

Systematic random sampling was used to select a total of 197 households (HHs) for tree/shrub inventory; of these, 91 were selected randomly for soil data collection based on their type and number of AF practitioners. Systematic random sampling is a type of probability sampling methods in which sample members from a larger population are selected according to a random starting point but with a fixed, periodic interval. The plot size established randomly for inventory data was 20 × 20 m for home garden AF, 50 × 100 m for parkland AF, 10 × 10 m for woodlot AF and 10 × 50 for boundary plantations. Inside each of the larger plot, five nested 1 × 1 m (one at the middle and four in the corners) sub-plots were laid for soil sampling. The diameter at breast height (DBH) and height was measured for each tree in all plots. For multi-stemmed woody species such as *Ziziphus spina-christ*, each stem was measured separately and the equivalent diameter of the plant was calculated as the square root of the sum of diameters of all stems per plant [15]. A total of 273 composite soil samples from depths of 0–20, 20–40 and 40–60 cm were collected from each plot using soil auger for determination of SOC, and an additional 273 soil samples were collected using soil core sampler for bulk density. The samples were transported to Tigray Agricultural Research Institute, Mekelle Soil Research Center. The soil samples for SOC analysis were air-dried, ground, homogenized and sieved with a 2- mm mesh size sieve. The C content of the soil samples was determined using the Walkley–Black method [14]. Bulk density was determined using oven dry method [16].

#### Terminology

In this study *home garden* AF deals with the cultivation of multipurpose and multi-storied trees combined with crop or/and animal husbandry around homestead, *Parkland* is areas retained with scattered multipurpose trees occur on farmland with farmers preference and protection and there is no grass cover in the cultivated land of the parklands; because the herbaceous plants were weeded in winter, *Woodlots* are sole stands of tree species planted on farm land or degraded lands to produce fuel wood, construction and land rehabilitation. *Boundary* plantation denotes trees retained or planted deliberately on the farm boundary.

### Data analysis

Species diversity in different AF practices were determined using species richness, Shannon index of diversity (H') and Shannon equitability or evenness index (E) [17]. The important value index (IVI) of each species with DBH ≥ 2.5 cm was calculated as the sum of its relative abundance, relative dominance, and relative frequency [18].

Above ground biomass (AGB) and below ground biomass (BGB) were estimated using the general allometric equations of [19], which were developed for AF species of Kenya. Tree/shrub biomass was converted to C by multiplying the above-ground biomass by 0.5 [20]. SOC stocks (Mg ha^−1^) were determined following the procedure of [21]. Ecosystem carbon stocks were calculated by summing of biomass and soil C stocks.

1 $$AGB = 0.091\; \times \;DBH^{2.472} \;;\;{\text{R}}^{2} \; = \;0.977,{\text{n}} = 72$$

Where, AGB is the aboveground biomass (dry mass per tree in kg) and DBH is diameter at a breast height (cm).

2 $$BGB = 0.490\; \times \;AGB^{0.923} ;\;{\text{R}}^{2} \; = \;0.95;{\text{n}} = 72$$

Where, BGB (dry matter per tree in kg).

### Statistical analysis

First, all data were checked for normality (using Kolmogorov- Smirnov test) and equality of variance (using Levene’s test). The size and variation in tree/shrub diversity, biomass and SOC stock data were described by mean and standard deviation. One-way ANOVA was performed (α = 0.05) to test differences in stand structure and biomass and soil carbon stock between each of the four AF systems. For SOC stock, two-way ANOVA was used since soil depth were considered as study factor together with AF practices. Non-normal data (DBH, height, basal area, richness) were analyzed using non-parametric (Kruskal–Wallis) test. When significant difference was found between the AF practices, a pairwise comparison LSD test was made. SPSS Statistics software (version 21) was used for the statistical analysis [22].

## Results

### Characterization of the indigenous AF systems

The farmers were practicing 61.2% of Parkland AF followed by 19.4% rotational woodlots, 12.2% home garden and 7.1% boundary plantings as a main AF practices in the study area. Parkland dominated the lowland AF systems, but was uncommon in the highland part of the study area. Eight nine percent of woodlots AF practice were found in the highland agroecology.

### Woody species diversity

These indigenous AF systems contained a total of 59 species, belonging to 48 genera and 32 families (Table 2). Moreover, household woody species diversity, species richness, and evenness significantly differed (p < 0.05) between each indigenous AF practice (Table 3). The Shannon diversity index and evenness value of home garden AF were significantly higher than the other AF practices while the list was recorded on the woodlot AF practices. The most abundant (number of individual per plot) species in the home garden are *Eucalyptus Sps, Olea Africana,* and *Sesbania sesban;* whereas, *Faidherbia albida* and *Ziziphus spina-christi* are the dominant tree species of park land AF practices (Appendix 1).

**Table 2:** Identified species, genera and families of indigenous AF practices

AF practices	Species	Genera	Families
Boundary planting	11	8	7
Home garden	23	21	14
Park land	47	36	26
Woodlot	8	6	5

**Table 3:** Household woody species richness, Shannon diversity index (H') and evenness in the indigenous AF practices of Tigray Region, Ethiopia

TAF practices	Richness	H'	Evenness
Boundary planting	2.43 ± 1.16 ^ab^	0.57 ± 0.48 ^b^	0.51 ± 0.37 ^b^
Home garden	3.44 ± 1.55 ^b^	0.93 ± 0.42 ^c^	0.76 ± 0.23 ^c^
Parkland	3.10 ± 2.14 ^b^	0.62 ± 0.49 ^b^	0.51 ± 0.33 ^b^
Woodlot	1.53 ± 1.00 ^a^	0.20 ± 0.36 ^a^	0.21 ± 0.34 ^a^
p- value	0.001	0.006	0.001

Similar letter shows not significant difference and different letters indicate significance differences between AF practices at p < 0.05

The variation between indigenous AF practices in woody species density, dbh, and height was significant (p < 0.05) (Table 3). Basal areas of the households AF practice ranges from the higher value (17.08 ± 13.81 m^2^ ha^−1^) in the woodlot AF practice to the lower (1.52 ± 1.89 m^2 ^ha^−1^) value in the parkland AF practices. A significantly higher dbh value was recorded on the parkland as compared with the other AF practices (Table 4).

**Table 4:** Mean (± sd) woody species density, dbh and height of agroforestry practices in Tigray Region, Ethiopia

AF practices	Stem number (ha ^−1^)	DBH (cm)	Height (m)	BA (m ^−2^ ha^−1^)
Boundary planting	132.14 ± 114.48 ^b^	11.54 ± 6.37 ^a^	10.79 ± 4.48 ^c^	1.76 ± 2.11
Home garden	187.50 ± 150.18 ^b^	12.46 ± 8.54 ^a^	8.05 ± 3.84 ^b^	2.89 ± 1.95
Park land	34.67 ± 26.02 ^a^	18.05 ± 15.28 ^b^	5.65 ± 2.05 ^a^	1.52 ± 1.59
Woodlot	1809 ± 506.85 ^c^	11.01 ± 6.37 ^a^	11.42 ± 3.76 ^d^	17.08 ± 13.81
p- value	0.00	0.00	0.00	0.00

Kruskal Wallis Test ANOVA was conducted to evaluate mean differences between groups and followed by Mann–Whitney U test for multiple comparisons. Similar letter shows not significant difference and different letters indicate significance differences between groups at p < 0.05; ns not significant

### Carbon stock potential

Mean above and belowground biomass C stock and SOC stock by layer are shown in Table 5. Total aboveground biomass C stock ranged from 2.78 to 21.43 Mg ha^−1^ in the four smallholdings AF practices. While smallholding total belowground biomass C stock ranged from 1.26 to 9.70 Mg ha^−1^. The contribution of above and below-ground biomass carbon stock of the study AF practices varied insignificantly between parkland, home garden and boundary planting while the woodlot was significantly different from home garden AF practices (p < 0.05). The aboveground biomass carbon stock for woodlot was higher by 75, 75.4 and 87% than a home garden, parkland and boundary planting AF practices. Smallholding total biomass C stocks ranged between 7.79 and 31.12 Mg ha^−1^.

**Table 5:** Mean (± standard deviation; n = 4) biomass carbon, soil carbon (SOC) and agroforestry system total (total biomass plus SOC 0–60 cm) carbon stocks (Mg ha^−1^) for each of the four studied agroforestry practices) and results of 1-way ANOVAs (at α = 0.05)

C_ Stock	Home garden	Parkland	Woodlot	Boundary planting	p
AGB	5.36 ± 2.92 ^ab^	5.27 ± 4.36 ^b^	21.43 ± 8.84 ^c^	2.78 ± 3.55 ^a^	0.000
BGB	2.43 ± 1.32 ^ab^	2.38 ± 1.97 ^b^	9.70 ± 3.40 ^c^	1.26 ± 1.61 ^a^	0.000
AGB + BGB	7.79 ± 4.23 ^ab^	7.79 ± 4.24 ^b^	31.12 ± 12.82 ^c^	4.03 ± 5.15 ^a^	0.000
SOC 0–20 cm	42.98 ± 7.90 ^b^	29.77 ± 14.75 ^a^	41.43 ± 15.60 ^b^	43.64 ± 14.44 ^b^	0.001
SOC 20–40 cm	33.33 ± 7.18 ^b^	20.06 ± 9.13 ^a^	28.95 ± 11.78 ^b^	34.95 ± 10.17 ^b^	0.000
SOC 40–60 cm	28.05 ± 6.92 ^ab^	21.85 ± 13.48 ^a^	26.57 ± 10.29 ^ab^	33.78 ± 14.75 ^b^	0.014
SOC 0–60 cm	108.81 ± 27.70 ^b^	71.69 ± 26.33 ^a^	96.95 ± 31.16 ^b^	112.74 ± 32.58 ^b^	0.000

Similar letter shows not significant difference and different letters indicate significance differences between AF practices at p < 0.05

Smallholding SOC stock for the 0–60 cm layer ranged between 72 and 112 Mg C ha^−1^ with the 0–20 cm layer accounting between 39 and 42% and the 20–40 cm layer ranged between 27 and 30% (Table 5). In contrast to biomass C stocks, mean SOC stocks were highest in the boundary planting AF (113 Mg ha^−1^) and the lowest was recorded in parkland AF (72 Mg ha^−1^) practices.

**Fig. 2:**
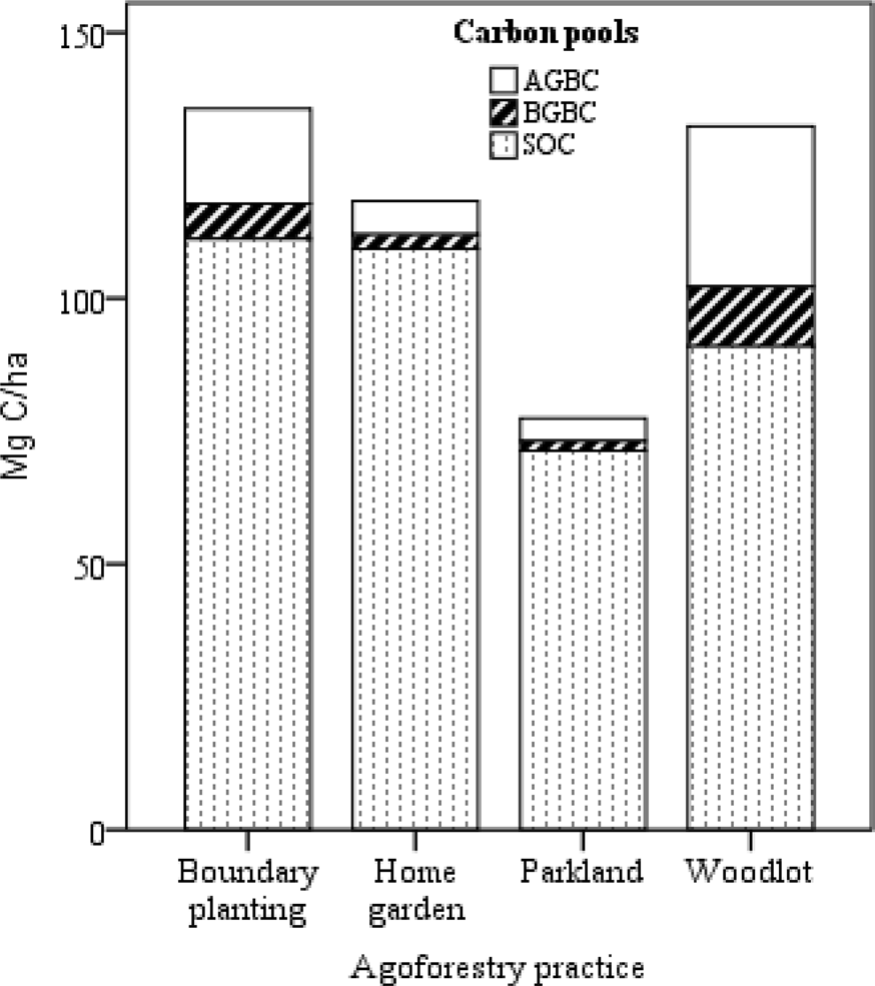
Total ecosystem (total biomass plus SOC 0–60 cm) carbon stocks (Mg ha−1) for each of the four studied agroforestry systems

Smallholding ecosystem C stocks ranged from 77 to 135 Mg ha^−1^ (Fig. 2). The ecosystem carbon stock estimated on the parkland AF was significantly lower than the other traditional AF practices (p < 0.05). The highest ecosystem carbon stock was recorded on the boundary planting and woodlot AF practices. The total biomass carbon stock accounts between 7 and 31% of the ecosystem carbon stock.

### Relationship between woody species diversity and carbon stock

Total biomass C stocks were significantly correlated with species abundance (Spearman r = 0.333; p = 0.002) but none of the biomass components were significantly correlated to the Shannon diversity index or species richness (Table 6). SOC stock for soil depth (0-60 cm) were significantly and positively correlated with the species richness and Shannon diversity index (Spearman r = 0.291 & 0.351; p < 0.01).

**Table 6:** Spearman correlations between biomass, soil carbon stocks, and woody species composition (n = 86)

Carbon stock component	Richness	Abundance	Shannon diversity index
AGB	− 0.192	0.308^**^	− 0.202
BGB	− 0.192	0.308^**^	− 0.203
TBC	− 0.183	0.333	− 0.197
SOC(depth, 0–60 cm)	0.291^**^	0.025	0.351^**^
Total AFC	0.065	0.209	0.115

AGB: Aboveground biomass; BGB: Belowground biomass; TBC: Total biomass carbon; SOC: Soil organic carbon; Total AFC: Total agroforestry carbon

^**^p < 0.01

## Discussion

### Woody species diversity

We documented high species richness in this sample of indigenous AF systems. This was similar with the species richness found in the Gedeo AF systems of Southern Ethiopia [9], but higher than the species richness found in the AF systems of South eastern Tigray and East Shewa, [23–25], and lower than the South central highlands of Ethiopia [26]. This could be due to environmental variability such as altitude, soils, topography, species adaptability and management strategy [9]. Besides to climatic factors the higher species richness in the lowland might be due to having large land holding size used to retain tree species and relatively lower human population density relative to the midland and highland agroecologies.

As compared to boundary plantings and woodlots, home garden AF demonstrated higher species diversity due to high richness and even distribution of species among small holdings in the highland areas. Similar findings have been reported in other Ethiopian highland areas [27]. The higher species richness observed in home garden is due to the fact that people in the highland areas plant large number of exotic and indigenous tree species in their farmland. Although the species evenness was a highest in midland-home gardens, woody species diversity was highest in parkland AF due to higher species richness and abundance. This is in line with pervious results from South-central Ethiopia [26].

### Carbon stock

From a biophysical point of view, the amount of carbon stored varies between different agroecologies and AF systems [28]. The mean total biomass carbon stock of smallholding farmers was within the range reported for the tropical African AF systems (12–228 t ha^−1^) [29, 30] and West Africa Sahel (0.7–54 t ha^−1^) [31] but higher than Kenya and Sri Lanka, respectively [32, 33]. Moreover, we find that carbon storage of indigenous AF in Tigray is lower than that of the Gedeo traditional AF system [34] and some systems of the tropics [35, 36]. This difference is attributable to variation in tree density, site characteristics, management type and variation of the use of biomass estimation models. Similarly, the higher biomass carbon stock found in our study woodlot AF practice was due to a higher number of stems per hectare.

In AF system, soil plays, a vital role in minimizing CO_2_ concentration in the atmosphere [37]. The SOC stocks in our studies are noticeably high compared to the biomass C stocks of other AF systems. [38] reported the SOC stocks from Agrisilviculture of Chhattisgarh, Central India is 27 Mg ha^−1^ on average for of 0–60 cm soil depth. The SOC stock of the 0–100 cm layer for the *Faidherbia albida* parkland in Segou, Mali has been reported 33.3 Mg ha^−1^ [31] and 43 Mg ha^−1^ for semi-arid *Acacia etabica* woodland in southern Ethiopia [39]. In contrast, the average SOC stock of our studied AF system was lower than the SOC stocks of the 0–60 cm layer for tropical forest which ranged from 121 to 123 Mg ha^−1^ [34]. This variation was due to variation in tree and stand variables (age, crops, tree diversity, composition and tree density), agroecological condition (altitude, climate and wind), soil characteristics (texture, fertility, physical, chemical and biological conditions) and management (fertilization, tillage, residues, land holding size and harvesting regime) [40, 41].

The boundary planting and home garden AF practices had the highest SOC stock, while the parkland AF system accounts for the lowest SOC stock. Boundary planting's higher SOC stock is attributable to the presence of herbaceous species and reduced soil disturbance relative to parkland. Cultivation of land with a higher level of soil disturbance might cause aggregate breakdown releasing of SOC formerly protected inside soil aggregates [41, 42]. Moreover, herbaceous and belowground fiber roots growing under boundary and home garden AF system contribute to increase carbon input to the soil and the buildup of soil organic matter [43]. In contrast, there is no grass cover in the cultivated land of the parklands; because the herbaceous plants were weeded in winter during soil cultivation and exported during the dry season.

The distribution of C stocks between soil and biomass varies among ecosystems and with the AF system. Park land accounts for the lowest (70 Mg C ha^−1^), ecosystem carbon stock of the four AF systems, although this figure is higher than the same agroecology of semiarid Zone in Senegal (52 Mg C ha^−1^) [44], in the parkland AF system of western Tigray (47.59 Mg C  ha^−1^), the exclosures of Tigray region in Northern Ethiopia (61.3 Mg C ha^−1^) [45], semi-arid (19 Mg C ha^−1^), sub-humid and humid (21 Mg C ha^−1^) and temperate (63 Mg C ha^−1^) ecozones [46]. Apart from the recorded high total C stock, a higher amount of carbon stock was found in the soil of the AF practices. The SOC (0–60 cm) to total biomass C stock ratio averaged 13.9 for the home garden, 9.2 for parkland, 3.1 for woodlot and 8.4 for boundary planting AF practices. The SOC stock to biomass C ratio in AF practices is affected by several factors including the age of the AF system that has been being practiced [47], types of tree species included and rotation age [12, 35], soil type [35], elevation, climatic condition [48, 49], and silvicultural management [45, 50].

Currently, carbon sequestration and biodiversity conservation are the most fundamental global environmental challenges, particularly in drylands. In the AF systems we examined, there was a negative but insignificant correlation between species richness and abundance on the one hand, and above- and belowground carbon stocks on the other. These results conflict with the positive correlation reported by [51].

We report that SOC stock increases with increasing species richness and Shannon diversity index in all four AF system. This is in keeping with the conventional wisdom that ecosystems with high tree diversity sequester more carbon in the soil than those which have lower diversity [52]. For example, [45] showed that SOC stock positively and significantly correlated with species richness and [53] found that species richness correlated to SOC in the home gardens of Kerala, India. However, the results of our study were in contrast with [51] which found a negative relationship between SOC stock (depth = 0–60 cm) and woody species diversity (species richness and Shannon diversity index). These differences might be related with species diversity in the AF system, management practices, age and site factors.

## Conclusion

In addition to the provision of food, the productive and protective function of trees, the Tigray regional state of Ethiopia's indigenous AF practices are critical for the mitigation of climate change and the conservation of tree diversity. Overall, 59 species from 48 genera and 32 families were found in the AF systems we surveyed. Compared to woodlot AF systems, greater species richness was recorded in the home garden and parkland small household AF system. Similarly, we found the highest Shannon diversity and evenness in home gardens. In addition, our analysis shows that, relative to other AF systems, rotational woodlots account for significantly (p < 0.05) greater biomass carbon stock. Second, we found that the method of boundary planting stored the highest total amount of SOC stock, followed by home garden and woodlot AF, respectively. We observed that there is a synergy between SOC stock and woody species diversity (i.e. species richness and Shannon diversity). The ecosystem C stock of these indigenous AF systems from in the semi-arid zone of Tigray is comparable to, and in some cases substantially higher than, those of tropical forests and other AF systems.

In general, indigenous AF systems have multiple advantages in improving the resilience of small-scale farmers by preserving tree diversity and mitigating climate change. In addition, the increased demand in Ethiopia and other sub-Saharan African countries for fuel wood and timber production, which are the key drivers of deforestation, can be met by indigenous AF systems and practitioners. AF will also contribute to the goal of reducing emissions from deforestation and forest degradation plus (REDD +) and conservation of tree diversity.

## Data Availability

The datasets used during and/or analyses the current study available from the corresponding author on reasonable request.

## Appendix 1

See Table

**Table 7:** List of woody species in the indigenous agroforestry system

No	Scientific name	Family	Local name	Number of individuals			
				BP	HG	PL	WL
1	*Acacia abyssinica Hochst.ex Benth.*	Fabaceae	Chea	5	8	1	7
2	*Acacia **etbaica*	Fabaceae	Seraw	2	–	253	–
3	*Acacia polyacantha*	Fabaceae	Gomero	–	–	1	–
4	*Acacia saligna*	Fabaceae	Akacha	3	1–	18	2
5	*Acacia senegal*	Fabaceae	Tsifri dimu	–	–	1	–
6	*Acacia seyal*	Fabaceae	keyih chea	–	–	123	–
7	*Acacia **sieberian*	Fabaceae	Tseada chea	–	–	13	–
8	*Acacia **torilis*	Fabaceae	Karwera	1	–	6	3
9	*Adansonia digitata*	Bombacaceae	Dima	–	–	1	–
1-	*Albezia amara*	Fabaceae	Sebqana	–	–	112	–
11	*Balanites aegyptiaca*	Balanitaceae	Mekie	–	–	2	–
12	*Boscia **angustifolia*	Capparaceae	Shisha	–	–	3	–
13	*Calotropis procera*	Asclepiadaceae	Ghindae	–	–	4	–
14	*Carissa **spinarum*	Apocynaceae	Agam	–	–	4	–
15	*Casuarina equisetifolia*	Casuarinaceae	Shewshewe	2	–	1	–
16	*Chamaecytisus proliferus*	Fabaceae	Tree lucern	3	–	–	–
17	*Citrus aurantifolia*	Rutaceae	Lomin	–	–	1	–
18	*Combretum adenogonium*	Combretaceae	Alenguata	–	–	2	–
19	*Combretum molle*	Combretaceae	Weyiba	–	–	81	–
2-	*Commiphora habessinica*	Burseraceae	Anqwa	–	–	11	–
21	*Cordia **africana*	Boraginaceae	Awhi	4	–	7–	–
22	*Cupressus lusitanica*	Cupressaceae	Tsihdi Ferenji	14	4	–	8
23	*Dalbergia melanoxylon*	Fabaceae	Zbbe	–	–	2	–
24	*Delonix regia*	Fabaceae	Diredawa zaf	–	–	–	1
25	*Dichrostachys cinerea*	Fabaceae	Kenney	–	–	1	–
*26*	*Dodonaoea angustifolia **L.f**.*	Sapindaceae	Tahses	1	1	–	1
27	*Dovyalis abyssinica*	Flacourtiaceae	Caito	–	–	7	–
28	*Eucalyptus **camaldulensis*	Myrtaceae	Keyih qelamintos	13	176	14	9
29	*Eucalyptus globulus*	Myrtaceae	Tsaeda–kelamitos	5–	369	–	115
*3-*	*Eucleara cemosa*	Ebenaceae	Kiliaw	–	–	6	–
31	*Faidherbia albida*	Fabaceae	Momena	2	–	239	–
32	*Ficus sycomorus*	Moraceae	Sagla	–	–	1	–
33	*Ficus vasta*	Moraceae	Daero	–	–	1	–
34	*Grevillea **robusta*	Proteaceae		1	–	2	8
35	*Jacaranda mimosifolia*	Bignoniaceae	Yetemenja zaf	2	–	1	–
36	*Juniperus procera*	Cupressaceae	Tse'hedi habesha	3	1	–	–
37	*Lannea fruticosa*	Anacardiaceae	Dgudgun	–	–	1	–
38	*Leucaena leucocephala*	Fabaceae	Lukina	–	–	6	–
39	*Malus domestica*	Rosaceae	Aple	1	–	–	–
4-	*Maytenus **senegalensis*	Celastraceae	Qebqeb	–	–	18	–
41	*Melia azadirach*	Meliaceae	Nim	–	–	3	–
42	*Moringa oleifera*	Moringaceae	Shiferaw	–	–	1	–
43	*Musa **paradisiaca*	Musceae	Muz	1	–	–	–
44	*Olea europaea subsp. Africana*	Oleaceae	Awlie	3–	–	1	16
45	*Ormocarpum pubescens*	Fabaceae	Alendia	–	–	2	–
46	*Osyridocarpus **schimperanus*	Santalaceae	Qortatimo	–	–	6	–
47	*Parkinsonia aculeata*	Fabaceae	Shewit hagay	–	–	6	–
48	*Populas **sps*	Salicaceae	Yekibret enchet	1	–	–	–
49	*Psidium guajava*	Myrtaceae	Zeitun	1–	–	9	–
5-	*Rhus glutinosa **A.Rich*	Anacardiaceae	Tetelo	–	–	2	–
51	*Schinus molle*	Anacardiaceae	Tikur berbere	2	1	–	3
52	*Securinega virosa*	Euphorbiaceae	Harmazo	–	–	1	–
53	*Sesbania sesban*	Fabaceae	Sesbania	16	–	–	–
54	*Stereospermum kunthianum*	Bignoniaceae	Adgi zana	–	–	25	–
55	*Tamarindus indica*	Fabaceae	Humer	–	–	1	–
56	*unknown*	–	Batequa	–	–	15	–
57	*Vernonia amygdalina*	Asteraceae	Grawa	5	–	–	–
58	*Ximenia americana*	Olacaceae	Meleo	–	–	2	–
59	*Ziziphus spina–christi*	Rhamnaceae	Gaba	–	–	999	–

7.

## Abbreviations

AF: Agroforestry
REDD^+^: Reduce emissions from deforestation and forest degradation plus
C: Carbon
SOC: Soil organic carbon
dbh: Diameter at breast height
AGB: Above ground biomass
BGB: Belowground biomass
